# Optimization of respiratory-gated auricular vagus afferent nerve stimulation for the modulation of blood pressure in hypertension

**DOI:** 10.3389/fnins.2022.1038339

**Published:** 2022-12-09

**Authors:** Ronald G. Garcia, Rachel Staley, Sarah Aroner, Jessica Stowell, Roberta Sclocco, Vitaly Napadow, Riccardo Barbieri, Jill M. Goldstein

**Affiliations:** ^1^Clinical Neuroscience Laboratory of Sex Differences in the Brain, Department of Psychiatry, Massachusetts General Hospital and Harvard Medical School, Boston, MA, United States; ^2^Innovation Center on Sex Differences in Medicine, Massachusetts General Hospital, Harvard Medical School, Boston, MA, United States; ^3^School of Medicine, Universidad de Santander, Bucaramanga, Colombia; ^4^Athinoula A. Martinos Center for Biomedical Imaging, Department of Radiology, Massachusetts General Hospital, Harvard Medical School, Boston, MA, United States; ^5^Scott Schoen and Nancy Adams Discovery Center for Recovery from Chronic Pain, Spaulding Rehabilitation Hospital, Harvard Medical School, Boston, MA, United States; ^6^Department of Gastroenterology and Center for Neurointestinal Health, Massachusetts General Hospital, Harvard Medical School, Boston, MA, United States; ^7^Department of Electronics, Information and Bioengineering, Politecnico di Milano, Milano, Italy; ^8^Department of Anesthesia and Critical Care, Massachusetts General Hospital, Harvard Medical School, Boston, MA, United States; ^9^Department of Medicine, Harvard Medical School, Boston, MA, United States

**Keywords:** blood pressure, heart rate variability, hypertension, race, sex, vagus nerve stimulation, transcutaneous vagus nerve stimulation

## Abstract

**Background:**

The objective of this pilot study was to identify frequency-dependent effects of respiratory-gated auricular vagus afferent nerve stimulation (RAVANS) on the regulation of blood pressure and heart rate variability in hypertensive subjects and examine potential differential effects by sex/gender or race.

**Methods:**

Twenty hypertensive subjects (54.55 ± 6.23 years of age; 12 females and 8 males) were included in a within-person experimental design and underwent five stimulation sessions where they received RAVANS at different frequencies (i.e., 2 Hz, 10 Hz, 25 Hz, 100 Hz, or sham stimulation) in a randomized order. EKG and continuous blood pressure signals were collected during a 10-min baseline, 30-min stimulation, and 10-min post-stimulation periods. Generalized estimating equations (GEE) adjusted for baseline measures were used to evaluate frequency-dependent effects of RAVANS on heart rate, high frequency power, and blood pressure measures, including analyses stratified by sex and race.

**Results:**

Administration of RAVANS at 100 Hz had significant overall effects on the reduction of heart rate (β = −2.03, *p* = 0.002). It was also associated with a significant reduction of diastolic (β = −1.90, *p* = 0.01) and mean arterial blood pressure (β = −2.23, *p* = 0.002) in Black hypertensive participants and heart rate in female subjects (β = −2.83, *p* = 0.01) during the post-stimulation period when compared to sham.

**Conclusion:**

Respiratory-gated auricular vagus afferent nerve stimulation exhibits frequency-dependent rapid effects on the modulation of heart rate and blood pressure in hypertensive patients that may further differ by race and sex. Our findings highlight the need for the development of optimized stimulation protocols that achieve the greatest effects on the modulation of physiological and clinical outcomes in this population.

## Introduction

Nearly one-third of the world’s adult population suffers from hypertension (Mills et al., 2016), including almost half (45%) of adults in the United States (Ostchega et al., 2020). Individuals with hypertension are at greater risk for developing cardiovascular disease (CVD), the number one cause of death worldwide (Virani et al., 2021). High blood pressure is also associated with numerous cognitive disorders, including dementia, and has become a leading contributor to the current global health crisis (Iadecola, 2014). While the front-line treatment for hypertension is pharmacological therapy with lifestyle modifications, most treated individuals continue to struggle with uncontrolled blood pressure levels and frequently need increased medication dosages or additional antihypertensive drugs (Lindholm, 2002; Thomopoulos et al., 2017). From 1980 to 2008, the worldwide population of patients with uncontrolled hypertension increased from 605 million to 978 million (or from 13.64 to 14.47% of the world’s population), because of population growth and aging (Danaei et al., 2011). The prevalence of treated but uncontrolled hypertension is higher among Black men and women, even after controlling for socioeconomic factors (Kramer et al., 2004). For these reasons, alternative preventative and therapeutic interventions for hypertension are desperately needed.

Previous research has demonstrated that cardiac autonomic imbalance and dysfunctional parasympathetic activity play an important role in the development and maintenance of hypertension (Grassi et al., 2011; Parati and Esler, 2012; Mancia and Grassi, 2014). Further, a reduction in heart rate variability (HRV), an index of parasympathetic function and cardiac vagal tone, has been associated with a poorer cardiovascular prognosis in patients with high blood pressure (Parati and Esler, 2012). Autonomic-mediated vascular dysfunction has also been proposed as the predominant mechanism underpinning the higher burden of hypertension among the Black population (Hill and Thayer, 2019). Importantly, some medications currently used for hypertension control, such as diuretics, have been associated with sympathetic activation (Grassi, 2016), which could result in undesirable effects and increased cardiovascular risk in subjects receiving this therapy. This highlights the need for alternative therapies for hypertension that are efficacious in the modulation of cardiac autonomic function.

The vagus nerve, is the longest cranial nerve in the body and is involved in the regulation of systemic parasympathetic activity, including effects on heart rate, blood pressure, and vascular resistance (Yuan and Silberstein, 2016). The vagus nerve contains efferent fibers that innervate cardiac muscle cells and the conduction system in the heart (Capilupi et al., 2020). An elevation in systemic blood pressure, sensed by arterial baroreceptors, results in increased vagal efferent traffic to the heart, causing the reduction of heart rate, ventricular contractility as well as HRV increase (Wehrwein and Joyner, 2013). Targeting the vagus nerve to regulate cardiac activity may be of significance in developing a novel treatment for hypertension that could have beneficial effects on reducing the risk for cardiovascular disease and comorbid disorders. Vagus nerve stimulation (VNS) via an implantable electrical stimulator has been approved by the US Food and Drug Administration (FDA) for epilepsy and treatment-resistant major depression and has shown some evidence of cardiac autonomic regulation (De Ferrari et al., 2011; Premchand et al., 2014; Nearing et al., 2016) and blood pressure reduction in animal studies (Plachta et al., 2014). Further, VNS has been evaluated as a treatment for heart failure in clinical trials and demonstrated the ability to significantly increase parasympathetic modulation and improve cardiac structure and function (Premchand et al., 2014, Nearing et al., 2021). Although VNS has exhibited encouraging results for cardiovascular regulation and blood pressure reduction, this surgical approach is highly invasive, expensive, and presents the risk of many serious side effects, including infection, pain, and vocal cord paralysis, reducing the appeal of this intervention (Daban et al., 2008).

There has been growing interest in a novel neuromodulation technique called transcutaneous auricular vagus nerve stimulation (taVNS), which electrically stimulates the auricular branch of the vagus nerve (ABVN). This approach has emerged as a potential non-invasive safer alternative to VNS (Ellrich, 2011). Anatomical studies have shown that afferent fibers from the ABVN terminate in the nucleus tractus solitarii (NTS) within the medulla (Nomura and Mizuno, 1984, Neuhuber and Berthoud, 2021). NTS modulates the activity of premotor cardiovagal neurons regulating peripheral vagal tone and cardiovascular function (Neff et al., 1998, Wehrwein and Joyner, 2013). Recent studies have shown beneficial effects of taVNS on the regulation of cardiovascular autonomic control (Sclocco et al., 2017; Staley et al., 2020; Carandina et al., 2021) and reduction of blood pressure levels in hypertensive patients (Fisher et al., 2018) without significant side effects. These findings provide a promising perspective for the evaluation of taVNS as a safe and effective antihypertensive intervention.

While taVNS is a promising technique for the modulation of cardiovagal activity and blood pressure levels in patients with hypertension, current stimulation parameters are based on historical VNS data (Thompson et al., 2021). Stimulation frequency has been described as a major parameter impacting the neuromodulatory effects of taVNS (Farmer et al., 2020). For instance, while many clinical applications of taVNS use 20–30 Hz stimulation, our group has found a significantly greater effect of high-frequency (100 Hz) stimulation on modulation of medullary vagal nuclei activity (Sclocco et al., 2020). Further, many of the brain regions modulated by vagal afference are morphologically and functionally sexually dimorphic (McEwen, 1983; Tobet and Hanna, 1997; Goldstein et al., 2001; Tobet et al., 2009), and therefore, taVNS actions could potentially be sex-dependent (i.e., an effect that is present in men and women but differs by sex), as suggested by previous publications (Yokota et al., 2022). Addressing these gaps in knowledge could provide valuable information for optimized stimulation protocols with improved clinical efficacy and compliance in hypertensive patients.

Finally, respiration may be a critical parameter for optimizing taVNS effects on the regulation of cardiovagal activity. The afferent input to NTS, and consequently its regulatory actions on peripheral vagal modulation, are affected by rhythmical oscillations in the respiratory cycle (Spyer, 1981; Gilbey et al., 1984; Neff et al., 2003; Dergacheva et al., 2010; Zoccal et al., 2014). Our group has shown that gating ABVN stimulation to exhalation, when NTS may be more receptive to afferent input, could optimize the modulatory effects of taVNS (Garcia et al., 2017a; Sclocco et al., 2019). Further, by supplying afference with intermittent, naturally irregular stimulation, respiratory-gated tVNS may also limit the neural habituation occurring with repeated stimulation over tens of seconds or even minutes, common with most VNS and taVNS applications (Zhou et al., 1997). This technique known as respiratory-gated auricular vagal afferent nerve stimulation (RAVANS) has been shown to significantly regulate vagal medullary nuclei activity, peripheral cardiovagal response and blood pressure levels in previous studies (Garcia et al., 2017b; Sclocco et al., 2017; Garcia et al., 2021). The objective of this pilot study was to identify frequency-dependent effects of our technique, RAVANS, on blood pressure and heart rate variability in hypertensive subjects and begin to examine if there are differential effects by sex/gender or race.

## Materials and methods

### Subjects

Twenty hypertensive subjects (54.55 ± 6.23 years of age; 12 females and 8 males) from a community-based sample were included in the study. Each participant was diagnosed with primary hypertension and was on stable doses of antihypertensive medications for at least 30 days prior to enrollment. Exclusion criteria included history of other cardio-, cerebro-, or peripheral vascular diseases, diabetes mellitus, morbid obesity (BMI >40 kg/m^2^), secondary hypertension, kidney or liver failure, thyroid disorders, pregnancy, traumatic brain injury with cognitive sequelae, psychiatric disorders involving psychosis, and metallic implants contraindicating taVNS. Additionally, we excluded individuals with a history of substance abuse within the past 12 months and individuals who had used recreational drugs (besides alcohol) within the month prior to screening. Patients were advised to avoid consuming any new medication throughout the study. No patients began a new antihypertensive regimen during the study.

All subjects gave written informed consent approved by an Institutional Review Board and the protocol was approved by the Human Research Committee of Massachusetts General Hospital. Subjects were compensated for their participation.

### Experimental protocol

**FIGURE 1:**
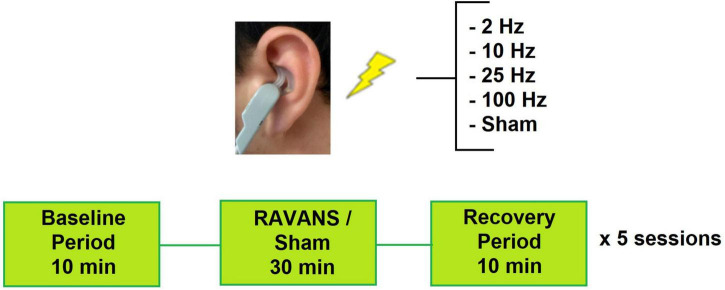
Experimental setup for study sessions. Subjects attended 5 sessions and receive one RAVANS frequency per visit.

The design of the study was a within-person experimental design of four different frequencies and sham stimulation in randomized order (Figure 1). Prior to the stimulation sessions, participants underwent a baseline evaluation with a brief medical history and physical assessment including height, weight, and blood pressure to confirm eligibility. During the active stimulation sessions, they received exhalatory-gated RAVANS in the left ear. Each session consisted of a 10-min baseline period, 30-min stimulation period, and 10-min post-stimulation period. Subjects were seated in an upright position for the entirety of the session. Participants received RAVANS at different frequencies for each session (i.e., 2 Hz, 10 Hz, 25 Hz, 100 Hz, or sham stimulation) in a randomized order. A minimum of 24-h was required between stimulation sessions to account for potential carry-over effects. All experimental sessions took place between 8 am and 11 am. All tests were performed in a quiet, dimly lit room at a comfortable temperature (21–23°C). Subjects were asked to abstain from caffeine, alcohol, tobacco, nicotine replacement products, and exercise within the eight hours leading up to the stimulation session. Participants were also instructed that they may eat up until three hours prior to the visit, consuming easy-to-digest foods and avoiding foods heavy in fat.

### Physiological data acquisition and analysis

Arterial blood pressure, electrocardiogram (ECG), and pulse rate signals were recorded continuously throughout each session. Blood pressure was collected using an inflatable finger cuff in conjunction with a Finometer device (Finapress Medical System, Netherlands). Electrocardiogram signals were acquired with electrodes placed on the chest using a Grass LP511 AC amplifier (Grass Technologies, Astro-Med, Inc. Product Group, RI, USA) and pulse rate was collected with a piezo pulse transducer (ADInstruments, CO, USA) placed on the subject’s thumb. All of these signals were collected at 400 Hz using a 16-channel PowerLab DAQ System on a laptop equipped with LabChart Data Acquition Software (ADInstruments).

Continuous blood pressure (BP) signals were semi-automatically annotated using the LabChart Pro Blood Pressure module (ADInstruments). BP peaks were automatically detected for the baseline, stimulation, and post-stimulation periods and then manually inspected and corrected if needed. Systolic (SBP), diastolic (DBP), and mean arterial blood pressure (MBP) values were extracted from the annotated data and used in statistical analyses. Heart rate (HR) and heart rate variability (HRV) measurements were also computed over the baseline, stimulation, and post-stimulation periods using LabChart (ADInstruments). Our primary HRV measurement was normalized power of the high-frequency band (HF power (%): HF/(HF + LF)). BP and HRV measures were averaged across each period.

### Respiratory-gated auricular vagus afferent nerve stimulation

Custom-built, ergonomically shaped Ag/AgCl electrodes were placed over the left cymba concha, a vagal-innervated auricular region, and stimulation was delivered with a UROstim transcutaneous electrical stimulator (Schwa Medico, Germany). Stimuli consisted of monophasic rectangular pulses with a pulse width of 300 μs and a duration of 1 s. Each subject’s respiration rate was captured through a pneumatic belt placed around their lower chest/abdomen. The respiration gating was implemented by sending the respiration signal to a laptop-controlled device (National Instruments USB DAQCard 6009, 14 bit i/o, with LabView 7.0 data acquisition software) and a computer algorithm detected the end-inhalation peaks in real-time to trigger the onset and offset of the stimulation. RAVANS was delivered during the exhalation phase with a 0.8 s delay between end-inhalation and the beginning of the stimulation. Stimulation amplitude was calibrated to an intensity that produced a moderate, non-painful sensation as indicated by a “5/10” on a subjective rating scale in which “0” represented no sensation and “10” represented a sensation that began to feel painful. For sham stimulation, subjects were told that stimulation intensity would be decreased to a level below their sensory threshold and the UROstim was gradually turned down before being turned off.

### Statistical analysis

Differences in demographic and clinical factors were examined by sex and race (White people, Black people, and Asian people/Pacific Islander). Categorical variables were compared using Fisher’s exact tests (as the majority of cells had expected counts <5) and continuous variables were compared by sex using t-tests and by race using ANOVA. Spearman correlations were used to examine relationships between BP and normalized HF power change variables.

Associations between RAVANS stimulation frequency and absolute change in HR, normalized HF power, SBP, DBP, and MBP from baseline to stimulation and baseline to post-stimulation were investigated using generalized estimating equations (GEE) (Liang and Zeger, 1986) adjusted for baseline measures. GEE models were implemented given that they account for intra-person correlation of the repeated measures of BP, HR and HF power. Absolute changes in BP, HR, and HF power were examined continuously (using an identity link in the GEE model). Average BP, HR, and HF power values from the full 30-min stimulation and the 10-min post-stimulation periods were used in primary analyses of change from baseline to stimulation, and baseline to post-stimulation, respectively.

To examine possible subgroup effects, analyses were stratified by sex and race (White people vs. Black people). Asian people/Pacific Islanders were excluded from subgroup analyses by race due to their limited numbers in our sample (*N* = 3). All analyses were performed in SAS 9.4 (SAS Institute, Cary, NC, USA).

## Results

### Participants

Study participants had a mean age of. 42.5 ± 9.2 years at hypertension diagnosis. Ten subjects were being treated via monotherapy and 10 subjects were receiving more than one antihypertensive medication at inclusion (Table 1). Female participants were significantly younger and had a greater body mass index (BMI) when compared to male subjects (Table 1). The race of participants was characterized by self-report (White people = 8, Black people = 9, Asian people = 3). No significant clinical differences were observed according to race at subject inclusion, except for the use of diuretics, which was significantly greater in Black participants (Black people = 88.9%, White people = 25%, Asian people = 0%; *p* = 0.006) (Table 2).

**TABLE 1:** Participant demographic and clinical characteristics by sex.

	All participants (*n* = 20)	Male (*n* = 8)	Female (*n* = 12)
Age (years)	54.5 ± 6.2	57.9 ± 4.6	52.3 ± 6.3*
Body mass index (kg/m^2^)	28.4 ± 3.6	26.2 ± 2.6	30.0 ± 3.5**
Time since diagnosis (years)	12.0 ± 7.9	11.7 ± 11.4	12.3 ± 5.6
**Race**			
White people	8 (40.0%)	4 (50.0%)	4 (33.3%)
Black people	9 (45.0%)	3 (37.5%)	6 (50.0%)
Asian people	3 (15.0%)	1 (12.5%)	2 (16.7%)
**Treatment**			
Monotherapy	10 (50.0%)	5 (62.5%)	5 (41.7%)
Multitherapy	10 (50.0%)	3 (37.5%)	7 (58.3%)
ACEIs	6 (30.0%)	3 (37.5%)	3 (25.0%)
ARBs	3 (15.0%)	1 (12.5%)	2 (16.7%)
Calcium channel blockers	9 (45.0%)	5 (62.5%)	4 (33.3%)
Beta blockers	1 (5.0%)	0 (0%)	1 (8.3%)
Diuretics	10 (50.0%)	2 (25%)	8 (66.7%)

ACEIs, angiotensin-converting enzyme inhibitors; ARBs, angiotensin II receptor blockers.

*p < 0.05 female vs. male; **p < 0.02 female vs. male.

**TABLE 2:** Demographic and clinical characteristics of participants by race.

	White people (*n* = 8)	Black people (*n* = 9)	Asian people (*n* = 3)
Age (years)	57.1 ± 5.1	54.4 ± 6.7	48.0 ± 3.0
Body mass index (kg/m^2^)	28.9 ± 4.0	28.1 ± 3.1	28.2 ± 5.1
Time since diagnosis (years)	14.5 ± 8.6	12.9 ± 6.5	3.3 ± 4.2
Sex (male)	4 (50%)	3 (33%)	1 (33%)
**Treatment**			
Monotherapy	3 (37.5%)	4 (44.4%)	3 (100%)
Multitherapy	5 (62.5%)	5 (55.6%)	0 (0%)
ACEIs	3 (37.5%)	1 (11.1%)	2 (66.7%)
ARBs	2 (25.0%)	0 (0%)	1 (33.3%)
Calcium channel blockers	6 (75.0%)	3 (33.3%)	0 (0%)
Beta blockers	0 (0%)	1 (11.1%)	0 (0%)
Diuretics	2 (25.0%)	8 (88.9%)*	0 (0%)

ACEIs, angiotensin-converting enzyme inhibitors; ARBs, angiotensin II receptor blockers.

*p = 0.006 Black people vs. White people.

### Respiratory-gated auricular vagus afferent nerve stimulation frequency-dependent effects on cardiovagal modulation

**FIGURE 2:**
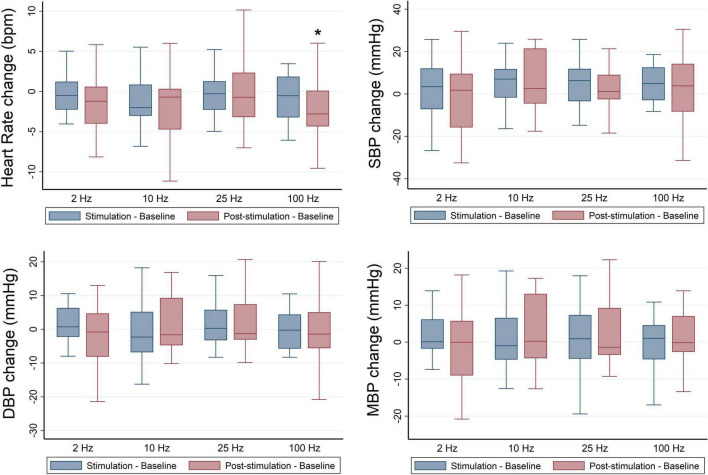
Frequency-dependent effects of RAVANS on the regulation of heart rate, systolic blood pressure (SBP), diastolic blood pressure (DBP), and mean blood pressure (MBP) in participants included in the study. Changes in variables estimated as absolute difference from baseline in comparison with sham stimulation. *p < 0.01.

No significant differences were identified for HR or normalized HF values at baseline across stimulation sessions (Table 3). Findings revealed a significant overall effect of RAVANS at 100 Hz in the reduction of HR following the stimulation period when compared with sham (β = −2.03, *p* = 0.002) (Figure 2). No significant changes were observed overall for other stimulation frequencies on HR or normalized HF (HFn) values during the stimulation or post-stimulation periods. However, a statistical trend toward significance was found for increased HFn during post-stimulation for RAVANS at 100 Hz when compared to sham (β = −4.51, *p* = 0.07).

**TABLE 3:** Baseline hemodynamic variables across stimulation sessions.*

	Sham	2Hz	10 Hz	25 Hz	100 Hz
Heart rate (bpm)	67.2 ± 10.1	70.1 ± 9.7	69.4 ± 9.9	71.6 ± 10.1	69.8 ± 9.5
HFn (%)	48.2 ± 21.1	52.5 ± 26.3	49.5 ± 22.0	51.4 ± 24.2	47.7 ± 22.5
SBP (mmHg)	135.6 ± 14.8	133.9 ± 16.3	132.8 ± 13.2	133.2 ± 17.1	130.8 ± 11.3
DBP (mmHg)	82.4 ± 11.1	83.8 ± 10.7	81.5 ± 11.4	82.1 ± 9.0	82.9 ± 9.0
MBP (mmHg)	100.1 ± 11.6	100.5 ± 11.3	98.6 ± 10.6	99.1 ± 10.7	98.9 ± 8.4

HFn, normalized HF power; SBP, systolic blood pressure; DBP, diastolic blood pressure.

*No significant differences in baseline cardiac measures across stimulation parameters.

**FIGURE 3:**
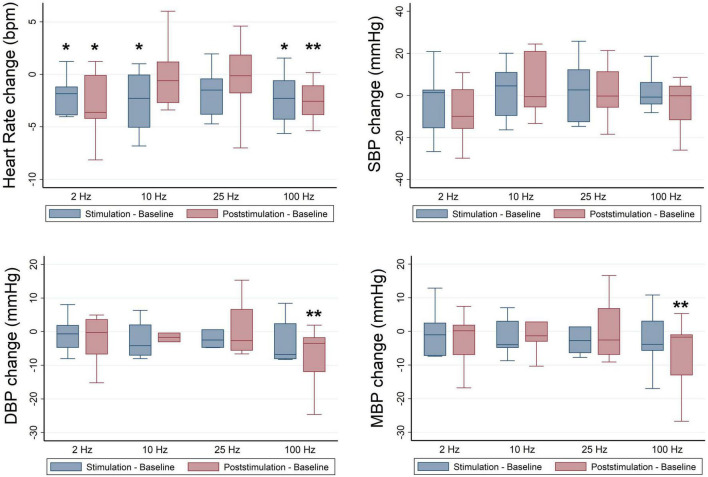
Frequency-dependent effects of RAVANS on the regulation of heart rate, systolic blood pressure (SBP), diastolic blood pressure (DBP), and mean blood pressure (MBP) in black participants included in the study. Changes in variables estimated as absolute difference from baseline in comparison with sham stimulation. *p < 0.05, **p < 0.01.

A subgroup analysis stratified by race revealed significant effects of RAVANS on the reduction of HR of Black participants at frequencies of 2 Hz (β = −1.32, *p* = 0.02), 10 Hz (β = −1.94, *p* = 0.01), and 100 Hz (β = −1.90, *p* = 0.01) during stimulation and for frequencies 2 Hz (β = −2.25, *p* = 0.04), and 100 Hz (β = −2.23, *p* = 0.002) during the post-stimulation period in comparison with sham (Figure 3). In sex-stratified analyses, RAVANS administered at 100 Hz significantly reduced the HR of female hypertensive subjects during the post-stimulation period when compared to sham (β = −2.83, *p* = 0.01). There was no statistical power to test for interaction effects of sex and race.

### Respiratory-gated auricular vagus afferent nerve stimulation frequency-dependent effects on blood pressure regulation

No significant differences were identified for BP values at baseline across stimulation sessions in the overall sample (Table 3), and there were no significant overall effects of RAVANS on the modulation of SBP, DBP, or MBP from baseline to stimulation or post-stimulation (Figure 2). However, analyses stratified by race revealed significant effects of RAVANS at a frequency of 100 Hz on the reduction of DBP (β = −1.90, *p* = 0.01) and MBP (β = −2.23, *p* = 0.002) of Black participants during post-stimulation (Figure 3). In addition, a statistical trend toward significance was found for a reduction in SBP during post-stimulation for RAVANS at 2 Hz when compared to sham (β = −8.26, *p* = 0.06). Stratified analyses did not reveal sex-dependent effects of RAVANS on the modulation of BP values, although we did not have sufficient power to test for interaction by sex. No significant correlations were identified between changes in BP values and normalized HF power in evaluated subjects.

## Discussion

The results of this pilot study suggest that taVNS gated to the exhalatory phase of respiration (RAVANS) has frequency-dependent effects on the modulation of hemodynamic variables in patients with hypertension. One 30-min session with RAVANS at a frequency of 100 Hz resulted in a significant reduction in heart rate in hypertensive patients when compared to sham, whereas lower frequencies did not show an effect. In addition, we identified significant modulatory effects of RAVANS-100 Hz on the reduction of HR, DBP, and MBP of Black hypertensive subjects and a suggestion of a differential sex effect on heart rate. These results highlight the potential for optimization and refinement of taVNS stimulation protocols for increasing its modulatory effects on the cardiovascular function of patients with hypertension, that may be dependent on sociodemographic characteristics.

Our findings are consistent with previous neuroimaging studies from our group that revealed frequency-specific effects of RAVANS on the modulation of medullary autonomic nuclei in healthy populations (Sclocco et al., 2020). In that previous study, RAVANS at 100 Hz was associated with greater activation of the NTS, nucleus ambiguous and locus coeruleus (LC), key components of the brainstem autonomic complex, when compared to other stimulation frequencies or sham stimulation. In addition, the activity of LC was positively associated with increased cardiovagal indices, suggesting that the administration of RAVANS at higher frequencies has an optimized effect on strengthening vagal afference to brainstem autonomic circuitry and is associated with increased efferent regulation of peripheral cardiovascular activity. Our results of taVNS frequency-dependent effects on the modulation of cardiovascular function are also consistent with a recent report from Yokota et al. (2022) that evaluated the effects of taVNS at different stimulation frequencies (1, 10, 25, and 100 Hz) on the autonomic response of 30 healthy participants. In this study, stimulation at 100 Hz had the most pronounced effect on the reduction of heart rate when compared to other frequencies.

The optimized effects of RAVANS at higher frequencies could be explained by the targeting of fast adapting, low-threshold mechanoreceptors of the ABVN near the dermal/epidermal junction (McGlone and Reilly, 2010). Receptors called Meissner corpuscles detect “cutaneous flutter” stimuli (5–50 Hz), while Pacinian corpuscles lie deeper within the dermis and respond to higher frequencies (50–400 Hz) of stimulation that is sensed as “vibration.” It has been suggested that the effects of transcutaneous stimulation of the ABVN are due to the stimulation of myelinated low-threshold A and B nerve fibers that serve mechanical purposes (Bermejo et al., 2017). Thus, from a physiological point of view, the administration of electrical pulses at a higher frequency range would be ideal for the activation of the myelinated ABVN fibers and transmission of vagal afferent signals (Aiyer et al., 2022). In addition, brainstem neurons receiving stimuli transmitted from auricular receptors can also respond in a frequency-dependent manner. For instance, studies have found that N-methyl-D-aspartate (NMDA) receptors in the NTS, activated by glutamate, contribute substantially to neurotransmission at frequencies >5Hz, but not at lower frequencies (Zhao et al., 2015). Such frequency-dependent physiology suggests that modulation of the vagal medullary complex and its regulatory actions on cardiovascular function, including blood pressure modulation, may indeed be optimized with higher stimulation frequencies.

Our results of greater RAVANS effects on Black participants with hypertension are intriguing and suggest differential effects of taVNS on the regulation of pathophysiological mechanisms of high blood pressure in this population. Black individuals are known to develop hypertension at an earlier age and have higher rates of resistance to treatment when compared to other racial/ethnic groups (Ferdinand and Townsend, 2012; Sim et al., 2013; Maraboto and Ferdinand, 2020). Likewise, this group has a higher prevalence of left ventricular hypertrophy and exhibits greater morbidity and mortality from complications of hypertension (Maraboto and Ferdinand, 2020). Several hypotheses (biologic and social) have been proposed to explain the differences in the manifestations and complications of hypertension in Black population. Biologic explanations include differences in pathophysiological mechanisms for hypertension, such as enhanced Renin Angiotensin Aldosterone system (RAAS) activity with exaggerated renal sodium retention and increased leptin-induced sympathetic nervous system activation (Spence and Rayner, 2018).

The mechanisms for greater reduction of HR, DBP, and MBP by RAVANS in Black individuals in our study could potentially be explained by the modulation of sympathetic nuclei in the medulla resulting in the downregulation of peripheral sympathetic drive. In response to increased vagal afference, NTS sends excitatory inputs to the caudal ventrolateral medulla, which then inhibits the rostral ventrolateral medulla, the main source of excitatory efferent output for the sympathetic nervous system (Wehrwein and Joyner, 2013). A direct measurement of sympathetic activity was not obtained in this study and therefore we cannot confirm this hypothesis. However, previous studies have found greater effects of taVNS on the modulation of cardiac autonomic function in those individuals with higher baseline sympathetic activity (Deuchars et al., 2018; Bretherton et al., 2019; Yokota et al., 2022).

In addition to potential differences in pathophysiological mechanisms for hypertension, our sample of Black participants also had a significantly higher use of diuretics, whereas White subjects had greater use of other antihypertensive medications. Most diuretics have been associated with increased sympathetic activity (Grassi, 2016), and therefore individuals receiving these medications may benefit from added interventions, like RAVANS, that regulate sympathetic drive. At the same time, the absence of significant effects on blood pressure regulation of White subjects in this study could potentially be explained by the lack of added benefit from RAVANS to sympathetic modulatory effects of the other antihypertensive medications that these subjects were already receiving. Finally, 80% of subjects on diuretics were female and all Black participants (except for one) were on diuretics. Thus, given the small sample size and variability (i.e., standard deviations) of the effects, there was no ability to unconfound the differential effects of sex or race from diuretic use on the differential impact of RAVANS frequency parameters. In addition, there were some significant differences in demographic characteristics between male and female subjects (i.e., age, BMI) that we were unable to control in the analysis due to our small sample size which may have confounded the sex-dependent effects observed in the study. These critical issues need further examination in future studies.

There are some limitations to this study. The main limitation of the study is related to the fact that in pilot studies like the one reported here, sample sizes are limited given initial funding limitations, which then limit the capability to detect other outcomes with small effect sizes and to test for differential effects by participant characteristics, such as sex and race, and thus subgroup results must be considered with caution. For instance, some of the observed changes in heart rate and HRV variables showed large variability, and therefore future studies with larger sample sizes and greater statistical power will need to refine and replicate these findings. Second, the experiment included variations in stimulation frequencies maintaining a constant pulse width, duration and a monophasic waveform. Since it has been reported that taVNS pulse width may also affect cardiovascular regulation (Badran et al., 2018), the combination of different frequencies and pulse width may result in distinct effects on blood pressure regulation. In addition, the use of different stimulation patterns, such as biphasic and triphasic waveforms, may also result in variations in stimulation efficiency (Kaniusas et al., 2020). Third, we used a fixed stimulation period of 1s during the respiratory phase of every respiratory cycle for all stimulation frequencies tested in the experiment. This resulted in a greater number of pulses (i.e., greater dosage) received with higher stimulation frequencies. Future experiments will need to control for this to evaluate whether the differential effect between high and low stimulation frequencies is due to the number of pulses received or to a different physiological response from ABVN receptors to the stimulation frequency. Finally, importantly, interactions between RAVANS and antihypertensive medications may also exist and will also need to be evaluated in future larger studies.

In conclusion, our study suggests that exhalatory-gated RAVANS at a frequency of 100 Hz effectively reduces heart rate in hypertensive patients and has significant effects on the modulation of blood pressure values in Black hypertensive subjects. Evaluation of this intervention in longitudinal studies with larger sample sizes will be required to address the generalizability of findings with respect to participant characteristics, i.e., race and sex, adjunctive antihypertensive medications, i.e., diuretics vs. others, and the duration of the intervention. Although our study was not designed as a clinical trial, the statistically significant rapid effects of RAVANS on modulation of the cardiovascular activity of hypertensive patients highlight the need for the development of optimized stimulation protocols that achieve the greatest effects on the modulation of physiological and clinical outcomes.

## Data availability statement

The raw data supporting the conclusions of this article will be made available by the authors, without undue reservation.

## Ethics statement

The studies involving human participants were reviewed and approved by the Partners Human Research Committee (PHRC), Massachusetts General Hospital, Partners Healthcare. The patients/participants provided their written informed consent to participate in this study.

## Author contributions

RG, VN, RB, and JG contributed to the conception and design of the study. RG, RSt, JS, and RSc participated in data collection and organization of the database. RG, RSt, JS, RSc, and RB participated in the processing and analysis of the physiological data collected. RG performed the clinical evaluation of participants and wrote the first draft of the manuscript. SA performed the statistical analysis. RSt, JS, SA, and JG wrote sections of the manuscript. All authors contributed to manuscript revision, read, and approved the submitted version.
